# Research status of risk factors and prevention of pancreatic cancer: A bibliometric and visual analysis

**DOI:** 10.1097/MD.0000000000041831

**Published:** 2025-03-14

**Authors:** Lichen Song, Guihua Wang, Ziyi Chen, Guangming Wang

**Affiliations:** a School of Clinical Medicine, Dali University, Dali, China; b Respiratory and Critical Care Medicine Department, The First Affiliated Hospital of Dali University, Dali, China; c Center of Genetic Testing, The First Affiliated Hospital of Dali University, Dali, China.

**Keywords:** bibliometrics, pancreatic cancer, prevention, risk factors, visualization

## Abstract

One of the biggest public health issues facing the globe today is pancreatic cancer (PC). To serve as a guide for clinically identifying existing research hotspots and conducting related studies in the future, bibliometric and visualization analyses of the literature on risk factors and PC prevention were carried out in this work. Results of published research from 2004 to 2024 were retrieved using the Web of Science database as a search platform. CiteSpace and VOSviewer were used for bibliometric and visual analysis. Based on the exclusion criteria, 868 articles in all were screened. Between 2004 and 2024, the quantity of articles published varied. Between 2017 and 2023, there was a consistent upward trend in the quantity of published literature. Cancer epidemiology biomarkers and prevention, cancers, and the Asian Pacific Journal of Cancer Prevention were the 3 journals with the most publications. The 2 nations with the most publications are China and the United States. The 2 nations with the most publications are China and the United States. The top 3 most published universities are Harvard University, the National Institutes of Health (NIH), and the National Cancer Institute (NCI). Buzzwords include body mass index, obesity, diabetes, smoking, and exercise.

## 1. Introduction

Pancreatic cancer (PC) is one of the extremely serious malignant tumors of the digestive system. In China, PC is the seventh most common malignant tumor in males and the eleventh most common in women. Its morbidity and mortality have been steadily rising in recent years, and they progressively rise with age.^[1]^ Low rates of early diagnosis, surgical resection, and medication efficacy are characteristics of PC. Due to the absence of distinct symptoms in the early stages of PC, the majority of patients are detected at an advanced stage, and even if some patients receive surgical resection, the recurrence rate is over 80% within 2 years.^[2]^ GLOBOCAN 2012 findings showed that new PC cases in China accounted for 19.45% of all newly diagnosed PC cases and 19.27% of all PC-related deaths globally.^[3]^

As we can see, PC has posed a serious risk to global health. Therefore, identifying high-risk persons and preventing PC require a thorough understanding of the risk factors for PC. The risk factors associated with the development of PC are diverse and complex, and studies have shown that PC morbidity and mortality are associated with poor lifestyle habits, such as smoking,^[4]^ drinking,^[5]^ obesity^[6]^ and dietary imbalance,^[7]^ these all raise the chance of PC. A higher risk of PC can also result from a number of other factors, including age,^[8]^ gender,^[9]^ blood group,^[10]^ family history, genetic history, race,^[11]^diabetes,^[12]^ chronic pancreatitis,^[13]^ intestinal infection,^[14]^ and more. In the modern world, PC is a significant public health issue that calls for a thorough examination of the risk factors for the disease.

In order to fully reflect the body of knowledge and development trends in a variety of research areas, bibliometrics is a quantitative literature analysis method that uses mathematics, statistics, and other scientific knowledge to examine the quantity, distribution, patterns, and relationships of the literature. Although reviews of independent risk factor analyses of PC have also been published, there are less comprehensive systematic reviews of risk factors and PC prevention, and few studies have used bibliometrics to generate in-depth analyses of this topic. To assess the current state and trends of risk factors and PC prevention, pertinent literature from 2004 to 2024 was selected. This work examines article features, author data, keywords, and risk factors in hot regions to identify information on highly published journals, highly published authors, current hotspots, and future advancements in the field that may be used as a reference for relevant researchers.

## 2. Methods

### 2.1. Sources of information and methods of search

**Figure 1.**
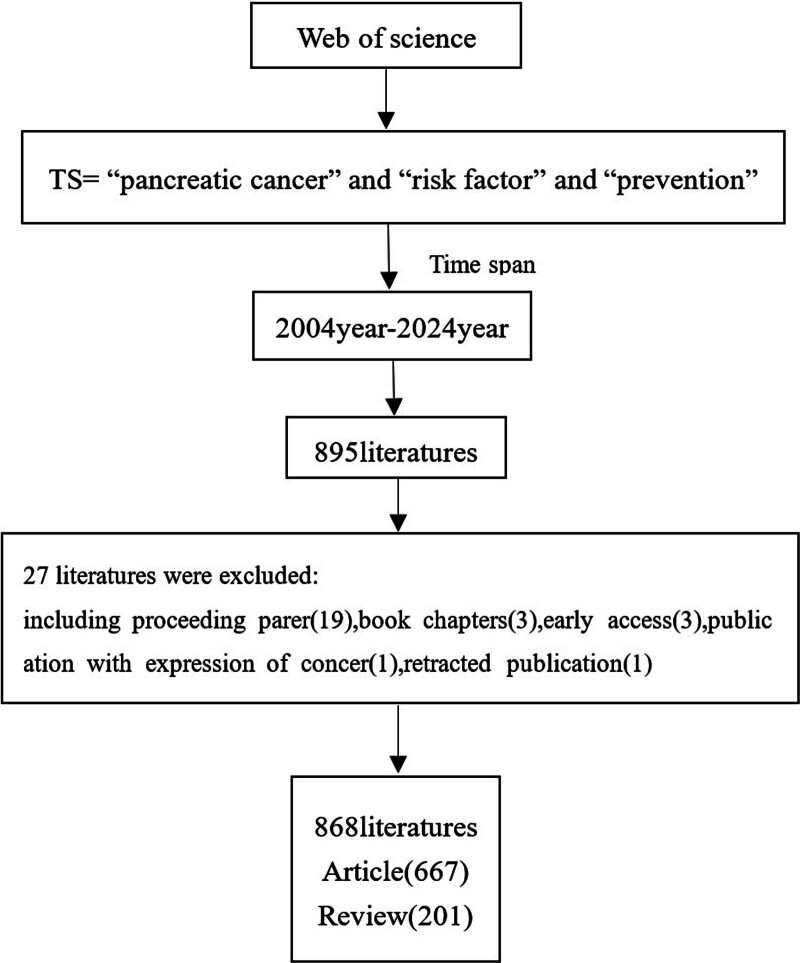
Diagram of paper search and screening process.

The literature on PC risk factors and prevention from 2004 to 2024 was found using the Web of Science (WoS) core collection platform. The following terms for searching were applied: (ALL = (PC) AND ALL = (risk factor) AND ALL = (prevention)) AND LA = (English) AND DT = (Article OR Review) AND DOP = (2004-01-01/2024-10-01), The time span was from 2004 to 2024. Criteria for inclusion: subjects in the literature pertaining to PC risk factors and prevention, which was published from January 11, 2004, to October 1, 2024. Exclusion criteria: non-article types. A total of 868 papers were eventually obtained (Fig. 1).

### 2.2. Bibliometric and visual analysis

We used the Web of Science (http://wcs.webofknowledge.com) to analyze the search results and describe all of the literature on PC risk factors and prevention. To analyze the data, we used CiteSpaceV6.3.R4 software. Set the number of years for each slice to 1 year in the parameter settings. As node types, we select “country,” “author,” and “keyword.” The “Pruning sliced networks” in “Pathfinder” were sliced as Pruning, and we set the selection criterion to *q*-index (*k* = 15) and TOP N = 50. The study hotspots for PC risk factors and prevention were summarized by utilizing VOSviewer (www.vosviewer.com) to construct a web visualization map based on data received from the Web Science Core Collection database and analyzing all keywords.

## 3. Result

### 3.1. A thorough examination of published articles

#### 3.1.1. Analysis of published articles’ quantity and trends

**Figure 2.**
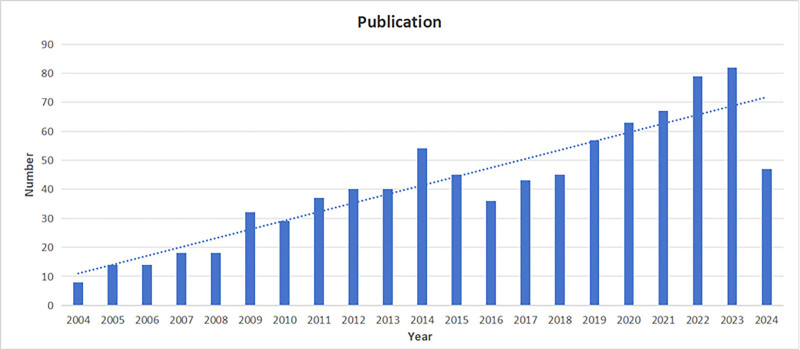
Trend chart of the number of published articles from 2004 to 2024.

There were 868 articles in all. A general upward trend was observed in the total number of published papers (Fig. 2) between 2004 and 2024. After 2014, there was a minor decline in the quantity of papers published. There are 2 distinct phases to the trend. Between 2004 and 2024, the quantity of articles published varied.2023 had the greatest increase in the quantity of published papers, with 82 being the highest number ever recorded. Between 2017 and 2023, there was a consistent increase in the quantity of published literature.

#### 3.1.2. Journal analysis

Analysis was done on the top ten journals with the most papers published. The top ten journals are listed in Table 1, along with details about their article counts and determining criteria. About 20% of the total number of articles in the topic have been published overall. The 3 journals with the most publications are Asian Pacific Journal of Cancer Prevention (2.7%), Cancers (3.2%), and Cancer Epidemiology Biomarkers & Prevention (11.6%).

**Table 1:** Risk factors and prevention of pancreatic cancer journal rankings (N = 868).

Rank	Journal	Publications	Percentage (n/N)	JCI	IF	JCR	Country
1	Cancer Epidemiology Biomarkers & Prevention	101	11.6	0.9	3.7	Q2	United states
2	Cancers	28	3.2	0.9	4.5	Q1	Switzerland
3	Asian Pacific Journal of Cancer Prevention	24	2.7	0.00	2.514	Q3	Japan
4	European Journal of Cancer Prevention	21	2.4	0.51	2.1	Q3	United states
5	Cancer Prevention Research	18	2.0	0.67	2.9	Q2	United states
6	International Journal of Cancer	18	2.0	1.21	5.7	Q1	Switzerland
7	Cancer Causes & Control	15	1.7	0.56	2.2	Q3	Netherlands
8	British Journal of Cancer	10	1.1	1.46	6.4	Q1	England
9	Cancer	9	1.0	1.29	6.1	Q1	United states
10	Nutrients	9	1.0	1.03	4.8	Q1	Switzerland

IF = impact factor, JCI = Journal Citation Indicator, JCR = Journal Citation Reports.

#### 3.1.3. Countries analysis

**Figure 3.**
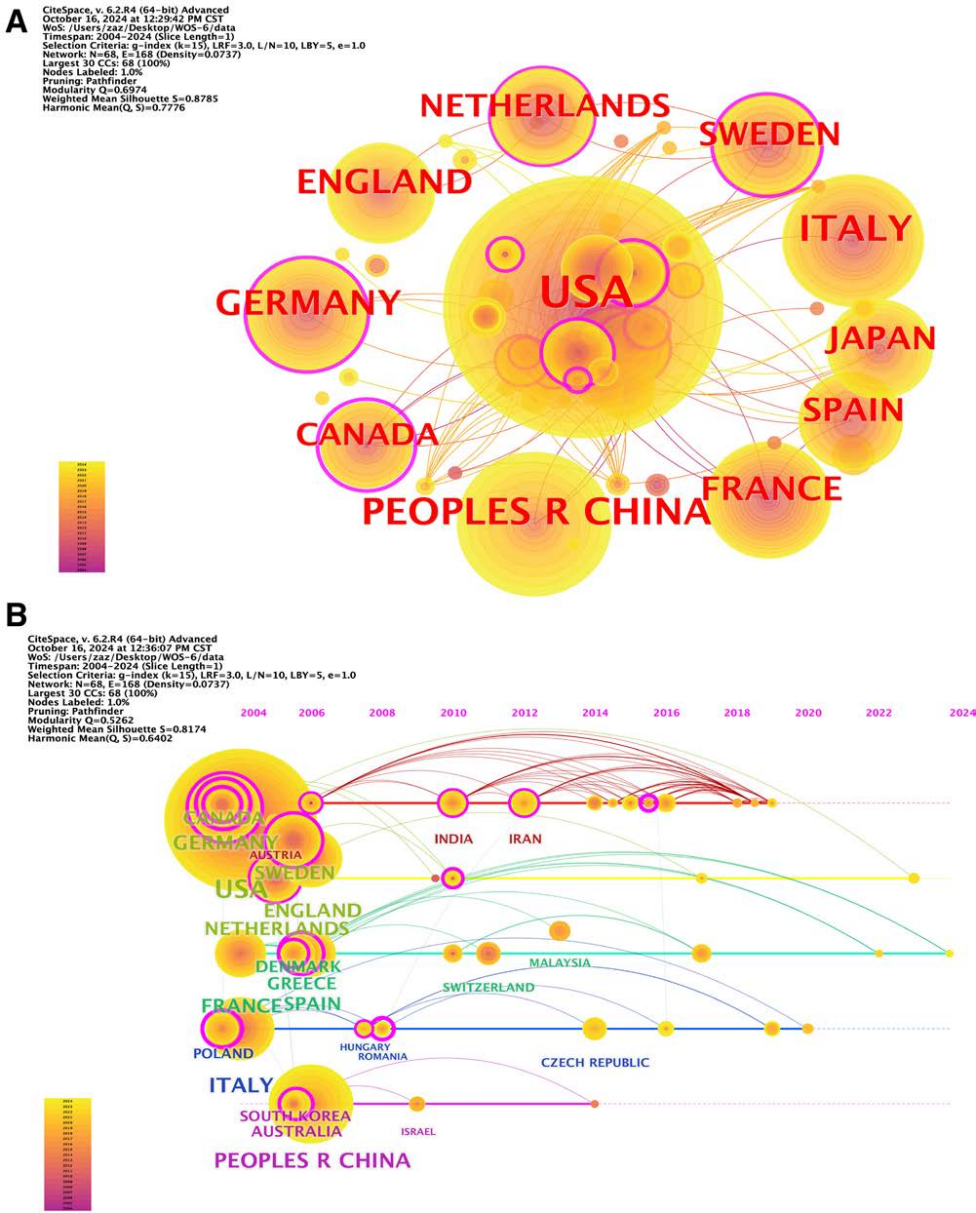
Network visualization map for top publication countries/regions. (A) CiteSpace was used to establish the top 10 countries and common clusters. (B) CiteSpace was used to analyze the clustering timelines of major countries in these 20 years.

As illustrated in Figure 3A, this overview examines the ten nations/regions with the greatest number of publications: the United States, China, Italy, the United Kingdom, France, Japan, Sweden, Spain, and the Netherlands. Using CiteSpace, we examined the clustering history of the major nations throughout the last 20 years. The United States and Italy are the primary nations in the earliest timeframe. These nations have greater cooperation ties and rigorous schedules. Figure 3B shows the specifics.

#### 3.1.4. Organizations analysis

The top 10 universities with the most publications are examined in this study. The primary organizations to which the writers belong are represented here. According to Table 2, the top 3 universities are the National Institutes of Health (NIH) in the USA (7.8%), Harvard University (8.5%), and the NIH National Cancer Institute (NCI; 7.7%).

**Table 2:** Ranking of institutions to which the first authors belonged (N = 868).

Rank	Institution name	Publications	Percentage (n/N)
1	Harvard University	74	8.5
2	National Institutes of Health (NIH) - USA	68	7.8
3	NIH National Cancer Institute (NCI)	67	7.7
4	University of Texas System	57	6.5
5	University of California System	54	6.2
6	World Health Organization	52	5.9
7	Helmholtz Association	52	5.9
8	International Agency for Research on Cancer (IARC)	52	5.9
9	German Cancer Research Center (DKFZ)	51	5.8
10	Harvard T.H. Chan School of Public Health	48	5.5

IF = impact factor.

#### 3.1.5. Citation frequencies analysis

**Figure 4.**
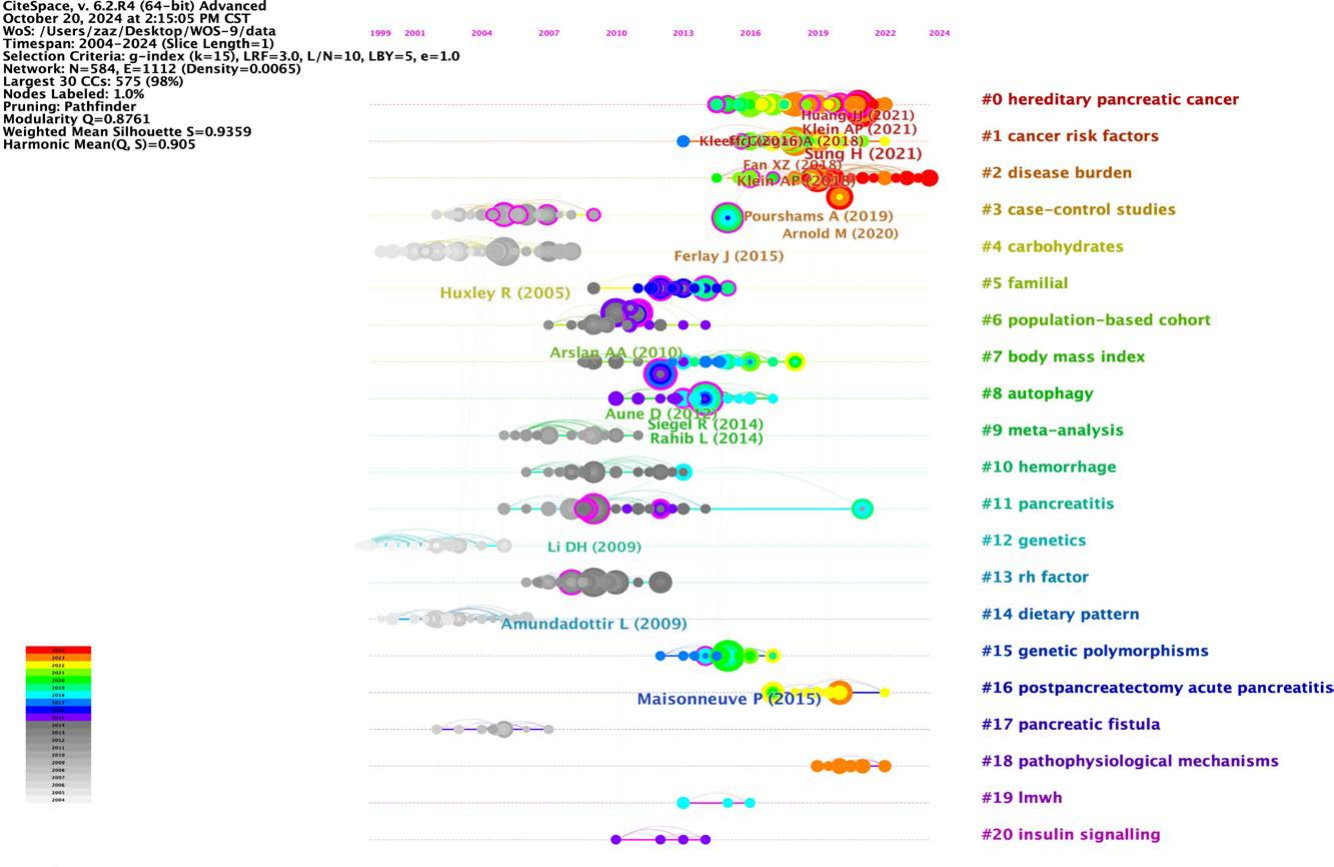
Timeline visualization of the reference co-citation map.

An article’s influence on a field of study can be gauged by the number of citations it receives. Table 3 displays the ten research papers (articles) that have received the most citations. and WoS takes into account the top 8 most cited. Table 4 displays the ten research papers (reviews) that have received the most citations. Co-cited references are regarded as an essential part of bibliometric studies. The frequency with which 2 texts are quoted simultaneously by another document is known as a co-citation.^[15]^ Because research clusters that are co-cited by numerous other publications arise when 2 papers in a field appear together, co-cited references serve as the foundation for articles in that field. The 10 referenced publications in the field, together with their first authors and journals, are included in Table 5. Figure 4 displays the top 10 cited papers from our analysis of the co-cited references using Citespace. Several clusters were created from the 868 articles that were obtained from WoSCC. Figure 4 displays the top 20 clusters in a vertical arrangement based on cluster size. The timeline is displayed horizontally. Since there has been a lot of research and collaboration in recent years, it is probable that many of the clusters may not extract characteristics adequately, even though the original clusters at the top of the picture are the most recent ones with higher reference or reference mutations. This may suggest the lack of novel mutations and indicates the constancy of research in this field.

**Table 3:** Top 10 most-cited research papers (article).

Title	First author	Total citations	Journals	Institution	Publication year	Country	IF
Burden of Gastrointestinal, Liver, and Pancreatic Diseases in the United States	Anne F. Peery	1613	Gastroenterology	University of North Carolina School of Medicine	2015	USA	25.7
Global Burden of 5 Major Types of Gastrointestinal Cancer	Melina Arnold	988	Gastroenterology	International Agency for Research on Cancer	2020	France	25.7
CANCER ETIOLOGY Stem cell divisions, somatic mutations, cancer etiology, and cancer prevention	Cristian Tomasetti	662	Science	Johns Hopkins University School of Medicine	2017	USA	44.7
Nadroparin for the prevention of thromboembolic events in ambulatory patients with metastatic or locally advanced solid cancer receiving chemotherapy: a randomized, placebo-controlled, double-blind study	Giancarlo, Agnelli	485	Lancet Oncology	University of Perugia	2009	Italy	41.6
Antidiabetic Therapies Affect Risk of Pancreatic Cancer	Donghui, Li	471	Gastroenterology	University of Texas System	2009	USA	25.7
Obesity and cancer risk: evidence, mechanisms, and recommendations	Ivana, Vucenik	407	Annals of the New York Academy of Sciences	University System of Maryland	2012	USA	4.1
The global, regional, and national burden of pancreatic cancer and its attributable risk factors in 195 countries and territories, 1990 to 2017: a systematic analysis for the Global Burden of Disease Study 2017	Akram, Pourshams	390	Lancet Gastroenterology & Hepatology	Tehran University of Medical Sciences	2019	USA	30.9
State of the epidemiological evidence on physical activity and cancer prevention	Christine M, Friedenreich	324	European Journal of Cancer	University of Calgary	2010	Canada	7.6
Trends and Patterns of Disparities in Cancer Mortality Among US Counties, 1980 to 2014	Ali H, Mokdad	299	JAMA-Journal of the American Medical Association	University of Washington	2017	USA	63.1
Insulin, glucose, insulin resistance, and pancreatic cancer in male smokers	RZ, Stolzenberg-Solomon	298	JAMA-Journal of the American Medical Association	National Institutes of Health	2005	USA	63.1

IF = impact factor.

**Table 4:** Top 10 most-cited research papers (review).

Title	First author	Total citations	Journals	Institution	Publication year	Country	IF
Epidemiology of pancreatic cancer	Milena Ilic	968	World Journal of Gastroenterology	University of Kragujevac	2016	Serbia	4.3
Obesity and cancer risk: Emerging biological mechanisms and perspectives	Konstantinos, I, Avgerinos	801	Metabolism-Clinical and Experimental	National & Kapodistrian University of Athens	2019	Greece	10.8
Adiposity and cancer at major anatomical sites: umbrella review of the literature	Maria, Kyrgiou	517	BMJ-British Medical Journal	Imperial College London	2017	England	93.6
Pancreatic cancer epidemiology: understanding the role of lifestyle and inherited risk factors	Alison P, Klein	498	Nature Reviews Gastroenterology & Hepatology	Johns Hopkins University	2021	USA	45.9
Leptin and cancer	Garofalo, C	473	Journal OF Cellular Physiology	Temple University	2006	USA	4.5
Global epidemiology and holistic prevention of pancreatitis	Maxim S, Petrov	441	Nature Reviews Gastroenterology & Hepatology	University of Pittsburgh	2019	USA	45.9
Coagulation and cancer: biological and clinical aspects	Falanga, A	284	Journal of Thrombosis and Haemostasis	Ospedali Riuniti di Bergamo	2013	Italy	5.5
Targeting Inflammation in Cancer Prevention and Therapy	Jelena, Todoric	280	Cancer Prevention Research	University of California System	2016	USA	2.9
Pancreatic cancer: A review of epidemiology, trend, and risk factors	Jian Xiong, Hu	234	World Journal of Gastroenterology	Putian University	2021	China	4.3
New mechanisms and the anti-inflammatory role of curcumin in obesity and obesity-related metabolic diseases	Adeeb, Shehzad	215	European Journal of Nutrition	Kyungpook National University	2011	South Korea	4.1

IF = impact factor.

**Table 5:** Top 10 co-citation of cited references.

Title	Country	Organization	Citation frequency	Journals	Publication year	First author
Global cancer statistics 2020: GLOBOCAN estimates of incidence and mortality worldwide for 36 cancers in 185 countries	France	World Health Organization	23	CA-A Cancer Journal for Clinicians	2021	Hyuna, Sung
Risk factors for pancreatic cancer: a summary review of meta-analytical studies	Italy	IRCCS European Institute of Oncology	22	International Journal of Epidemiology	2015	Patrick, Maisonneuve
Type-II diabetes and pancreatic cancer: a meta-analysis of 36 studies	Australia	University of Sydney	19	British Journal of Cancer	2005	R Huxley
Genome-wide association study identifies variants in the ABO locus associated with susceptibility to pancreatic cancer	USA	National Institutes of Health	19	Nature Genetics	2009	Laufey, Amundadottir
Anthropometric Measures, Body Mass Index, and Pancreatic Cancer A Pooled Analysis From the Pancreatic Cancer Cohort Consortium (PanScan)	USA	New York University	18	Archives of Internal Medicine	2010	Alan A. Arslan
Projecting Cancer Incidence and Deaths to 2030: The Unexpected Burden of Thyroid, Liver, and Pancreas Cancers in the United States	USA	University of Texas System	18	Cancer Research	2014	Lola, Rahib
Genome-wide meta-analysis identifies 5 new susceptibility loci for pancreatic cancer	USA	Johns Hopkins University	18	Nature Communications	2018	Alison P, Klein
Cancer Statistics, 2014	USA	American Cancer Society	17	CA-A Cancer Journal for Clinicians	2014	Rebecca, Siegel
Body mass index, abdominal fatness and pancreatic cancer risk: a systematic review and non-linear dose–response meta-analysis of prospective studies	England	Imperial College London	17	Annals of Oncology	2012	Aune
Pancreatic cancer epidemiology: understanding the role of lifestyle and inherited risk factors	USA	Johns Hopkins University	16	Nature Reviews Gastroenterology & Hepatology	2021	Alison P, Klein

### 3.3. Author analysis

#### 3.2.1. Principal investigators

The first authors to publish in this topic in the past 20 years are examined in this study. Table 6 lists the top 10 first authors by the quantity of papers they have published. Specifically, the 3 authors with the most publications are identified as Albanes, Demetrius, Duell, Eric J, and Stolzenberg-solomon, Rachael Z.

**Table 6:** Ranked by the number of articles published by the author(N = 868).

Rank	Authors	Publications	Percentage (n/N)
1	Albanes, Demetrius	19	2.1
2	Duell, Eric J	19	2.1
3	Stolzenberg-solomon, Rachael Z	18	2.0
4	Bueno-de-mesquita, H Bas	15	1.7
5	Boutron-ruault, Marie-Christine	14	1.6
6	Li, Donghui	14	1.6
7	Jacobs, Eric J	14	1.6
8	Tjonneland, Anne	13	1.4
9	Giovannucci, Edward L	12	1.3
10	Bracci, Paige M	12	1.3

#### 3.2.2. Co-authorship networks analysis

**Figure 5.**
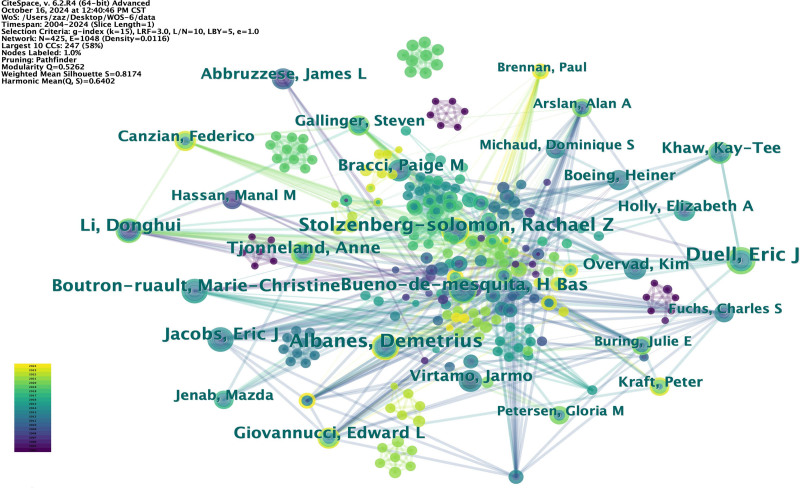
Author cluster co-occurrence analysis. Different colors represent different clusters, and the main clusters are labeled with the author name.

In this study, we use CiteSpace to analyze the state of collaboration between authors. Figure 5 shows the clusters of top authors in the field and the distribution of collaboration times. There are 8 main clusters divided according to the time indicated by the color of the lines, with 4 core clusters, and a number of smaller dispersed clusters.

### 3.5. Research trend analysis and keyword-based burst detection

**Figure 6.**
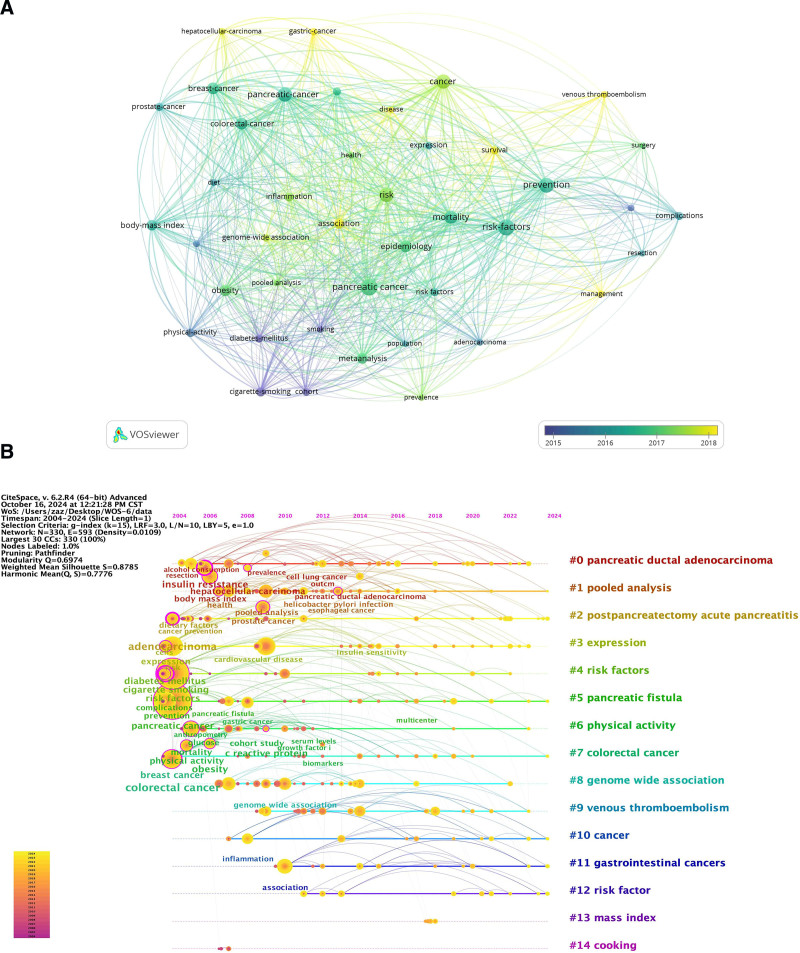
The keywords and hot topics during 2004 to 2024 were analyzed visually. (A) Co-occurrence analysis of the most popular keywords over the decade was performed using VOSviewer. (B) The time line analysis of hot words in the past 20 years is carried out by CiteSpace.

**Figure 7.**
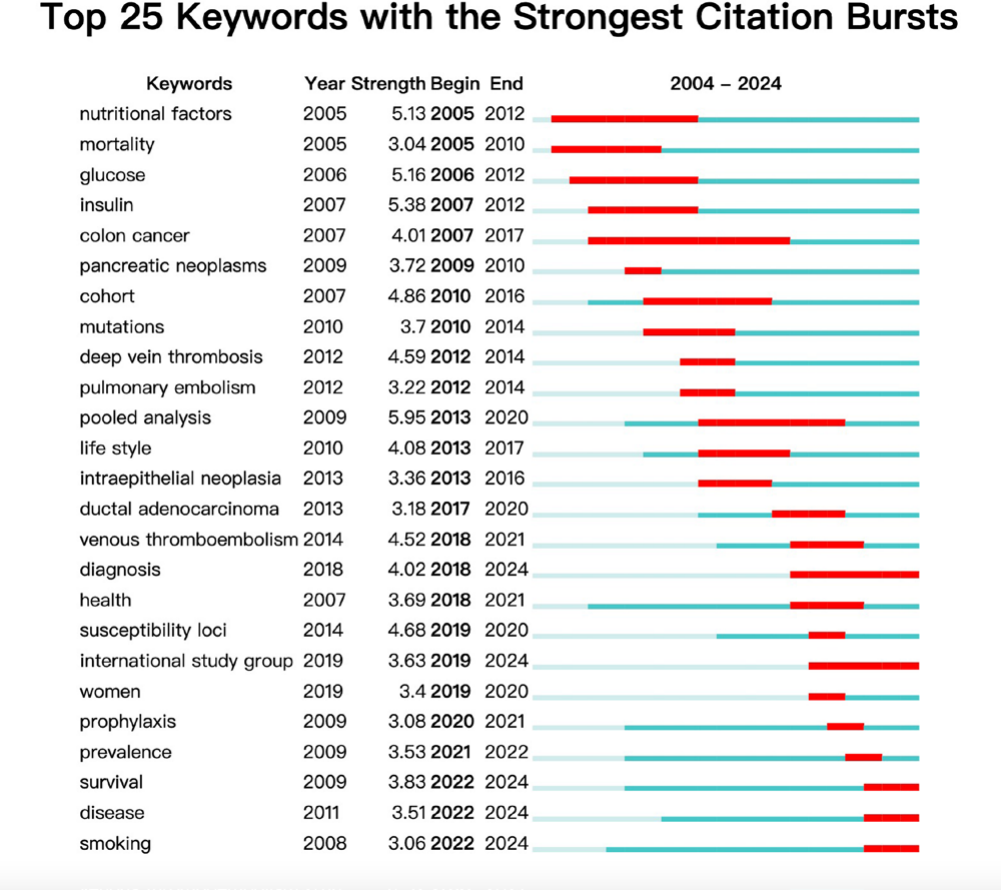
From 2004 to 2024, 25 keywords with high frequency emerged. Visual analysis of the outbreak intensity of 25 hot words with the highest outbreak rate in the last 20 years.

Research trends and hotspots can be inferred indirectly from keyword analysis. Keyword visualization (Fig. 6A), keyword timeline analysis (Fig. 6B), and visualization of epidemic intensity for the top 25 most common words with the greatest outbreak rate over the past 20 years (Fig. 7) were all conducted in this study using VOSviewr and CiteSpace. More common terms were cited in the preventative literature, and it was discovered that keywords like body mass index, smoking, exercise, obesity, and diabetes were risk factors for PC. In general, colon cancer has the highest frequency of outbreaks and the longest duration of research. With the maximum outbreak intensity of 4.01 times between 2007 and 2017, it has emerged as a research hotspot.

### 3.6. Examining the way the WoS discipline (subject) category evolved

**Figure 8.**
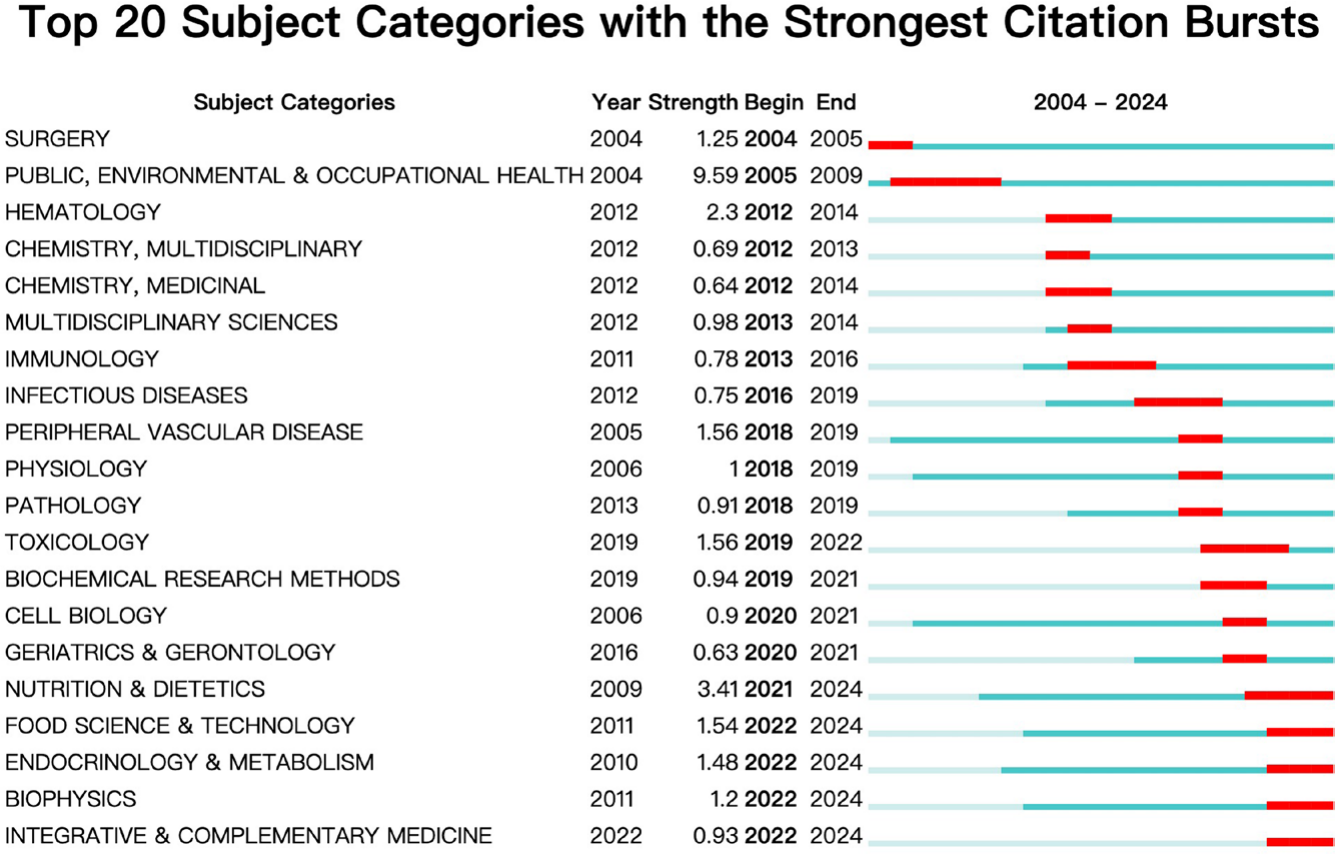
Subject categories with the strongest citation bursts.

From the standpoint of research direction, we analyzed mutations in the WoS subject (disciplinary) categories using CiteSpace. Figure 8 displays the top 20 subject categories with the most robust citation bursts. Between 2022 and 2024, the following categories saw the most recent mutations: “Food Science and Technology,” “Endocrinology and Metabolism,” “Biophysics,” and “Integrative and Complementary Medicine.” These results demonstrate recent shifts in the field’s focus.

## 4. Discussion

Researchers have worked to investigate the risk factors for PC and how to prevent it for the past 20 years. Additionally, the number of publications in this sector is growing annually. Using CiteSpace and VOSviewer software, this study examines research hotspots and trends in PC-related risk factors and prevention, as well as the top nations/regions, institutions, journals, authors, and keywords.

### 4.1. Features of publications on PC risk factors and prevention

Based on patterns in the quantity of publications in the subject, the field’s progress over the previous 20 years can be split into 2 stages. The year with the most publications and the quickest increase over this time frame was 2014. Since 2014, the frequency of PC-related deaths may have decreased due to improvements in imaging and surgical techniques, the adoption of new chemotherapy regimens, early tumor marker detection, and a better knowledge of the pathophysiology of PC.^[16]^ Because of this, a lot of scholars might have become more interested in this field in 2014, which is why the number of papers published that year grew the fastest. The number of publications in the other phase, which is one of rapid development and prevalence, is rising annually between 2015 and 2023. PC-related deaths are steadily rising. The field has expanded quickly throughout this time due to the growing epidemiological burden of PC^[11]^ as well as the widespread awareness of illness prevention in recent years and the merger of the public health and clinical fields.

Bibliometric techniques were used to analyze journal data in depth. With 425 publications, or 27.9% of the top 20 producing countries/regions, the United States in particular stood out as the most prolific nation in the field of PC risk factors and prevention in terms of the quantity of published articles. The People’s Republic of China (140, 9.24%), Italy (115, 7.6%), Germany (94, 6.2%), the United Kingdom (86, 5.7%), and France (78, 5.14%), came next. Over the course of the 5 years, the United States published more than other nations, indicating the ongoing interest in studying PC risk factors and prevention.

Considering the significance and leadership role of institutions in a certain discipline, we examined prominent organizations in PC risk factors and prevention. Of them, the United States is home to 6 of the top ten organizations. These findings demonstrate that the US has been a top producer of high-caliber and productive articles in PC risk factor and preventative research. The timeline graph (Fig. 3B) demonstrates that over the 20-year period from 2004 to 2024, Central Asian and some European nations have consistently produced positive outcomes. Both the disease burden and PC incidence are high in these nations. These nations have given the field of PC considerable thought and have made important advancements in it. The 3 organizations with the most publications are Howard University, the NIH, and the NCI. To jointly advance the growth of this field, institutions and nations can improve their cooperation and exchanges.

The extensive body of literature in this field has benefited greatly from the close collaboration of authors, according to the cluster analysis of authors. The most papers in this field are by Albanes, Demetrius, who specializes in cancer epidemiology, cancer control, and cancer prevention. Eric J. Duell is the second most prolific author of a study that examines a range of illness risk factors, including physiological and chemical components. Rachael Z. Stolzenberg-Solomon, the third most published researcher in the subject, focuses on the burden of disease and epidemiology of PC. Although the researchers’ areas of interest vary, it is evident that they are all looking at PC risk factors and prevention. Researchers from many fields can work together more effectively and provide more useful outcomes. In the meantime, we can focus more on the academic accomplishments of these experts if we wish to learn about the most recent developments in this field.

### 4.2. Current developments and patterns in the prevention and risk factors for PC

By using hotspot breakthrough analysis and keyword visualization, we discovered that bad lifestyle choices, biological factors, and disease states are the main risk factors for PC. Primary and secondary prevention are the main topics of PC preventive research. The prevalence of PC is influenced by negative behaviors and lifestyle choices, such as unhealthy eating patterns. PC is just one of many malignant tumors for which smoking and drinking alcohol are risk factors. According to research conducted by the International Agency for Research on Cancer, current smokers are at least 100% more likely to develop PC than nonsmokers, and the risk rises as cigarette use and smoking duration increase. According to studies, patients who drink excessively have a markedly higher risk of PC; this risk is especially high for frequent drinkers who are men.^[4]^ Body mass index (BMI) > 25.0 kg/m^2^ raises the risk of PC, and obesity has been shown to increase morbidity and mortality from PC. An improper diet may cause PC since the pancreas secretes pancreatic juice to aid in food digestion and absorption.^[6]^

Human tumors can be caused by a variety of biological processes. According to certain research, the incidence of PC rises with age, making age an independent risk factor for the disease’s development.^[17]^ It has been observed that the male population has 1.46 times the incidence and mortality rates of PC compared to the female population.^[9]^ Compared to whites and yellows, black people have greater incidence and mortality rates of PC.^[18]^ People with non-O blood types are more likely to acquire PC, according to a number of domestic and international research that have established a link between blood type and PC development.^[19]^ The development of PC is influenced by mutations in the KRAS gene, and 5% to 10% of PCs are linked to heritable genetic alterations.^[20]^ PC has a familial tendency.

Intestinal dysbiosis, infections, and chronic pancreatitis are among the disease variables that raise the risk of PC. The risk of PC is increased by long-term diabetes, and patients with type 1 or type 2 diabetes mellitus have a 1.8 to 2 times higher risk of PC than the general population.^[21]^ Research has indicated that individuals with chronic pancreatitis are around 5% more likely than the general population to develop PC, which is a significant 13-fold increase in risk.^[22]^ Numerous bacteria have been linked to the development of PC; the risk of PC is increased by Helicobacter pylori infection, increased by Porphyromonas gingivalis, and lowered by Streptococcus digestive tract.^[23]^ In addition to etiology research, hot themes like health management, survival rate, follow-up, and outcomes also surfaced, according to the results of the keyword and hot topic analysis. It is evident that the current focus on cancer has gradually shifted from clinical treatment to prevention, management, and improving the quality of life for cancer patients. This underscores the necessity of enhancing prevention strategies and putting in place long-term cancer control measures. Achieving positive health outcomes and lowering healthcare costs are significantly impacted by effective PC prevention. Investigating the risk factors for PC is essential to putting preventative measures into place, as the first step in prevention is to identify the risk variables linked to cancer risk.

According to the risk factor analysis above, changing one’s behavior and way of life is the major way to prevent PC. Tobacco control must be implemented for smoking, and both pharmacological and non-pharmacological therapies can be used to achieve this. In order to prevent chronic diseases, secondary prevention is equally crucial. Since chronic infections are typically asymptomatic, screening should be prioritized more. The government should speed up the screening of high-risk groups and increase public and physician awareness.

### 4.3. Study limitations

There are certain restrictions on this study as well. The literature database for this study is still lacking because it was restricted to Web of Science. Second, as academic journals still prefer to publish in English, we only looked at papers written in that language, leaving out publications written in other languages.

## 5. Conclusion

This study uses bibliometrics and visualization software analysis to show the state of research on PC risk factors and prevention worldwide. The impact of biological, behavioral, and environmental factors on PC, as well as primary and secondary prevention, are the main topics of current research. There are still a lot of unknown elements that contribute to PC development, and better screening programs and PC prevention techniques need to be investigated.

## Author contributions

**Conceptualization:** Lichen Song, Guihua Wang.

**Data curation:** Lichen Song.

**Formal analysis:** Lichen Song, Guihua Wang.

**Funding acquisition:** Guangming Wang.

**Investigation:** Lichen Song, Ziyi Chen.

**Methodology:** Lichen Song, Guihua Wang, Ziyi Chen.

**Software:** Lichen Song, Guihua Wang.

**Supervision:** Guangming Wang.

**Visualization:** Lichen Song.

**Writing – original draft:** Lichen Song.

**Writing – review & editing:** Lichen Song, Guihua Wang, Guangming Wang.
